# Examining the effects of competitive state anxiety and goal orientation on sports performance of college track and field athletes

**DOI:** 10.3389/fpsyg.2025.1607747

**Published:** 2025-06-13

**Authors:** Ziwei Wu, Feng Luo, Xu Liu, Junhua Li, Yin Zhou, Jie Luo

**Affiliations:** ^1^School of Educational Science, Hunan Normal University, Changsha, China; ^2^College of Sports and Health, Huaihua University, Huaihua, China; ^3^School of Educational Science, Huaihua University, Huaihua, China; ^4^School of Physical Education, Hunan First Normal University, Changsha, China

**Keywords:** competitive state anxiety, goal orientation, sports performance, track and field athletes, task orientation, ego orientation

## Abstract

Existing studies on athletes’ sports performance (SP) have identified significant associations between competitive state anxiety (CSA) and goal orientation (GO). However, few studies have explored the intrinsic relationships among CSA, GO, and SP. Moreover, there is a lack of attention paid to college track and field (T&F) athletes as a specific group, particularly regarding the role of GO in this process. We investigated the relationship between CSA and GO as well as their effects on athletes’ SP using a sample of Chinese college T&F athletes (*N* = 87, 44.8% female). Specifically, the mediating role of GO in the relationship between CSA and SP was examined. Task orientation significantly mediated the relationship between self-confidence and SP, with higher levels of task orientation leading to shorter completion times for the 1500-meter race. However, ego orientation was not associated with sports performance. These findings suggest that fostering task orientation and enhancing self-confidence are crucial to improving SP. This study deepens our understanding of CSA and GO in athletes by contributing to the current theoretical framework. Furthermore, our results offer practical recommendations for coaches and educators to develop evidence-based training strategies aimed at optimizing athletes’ psychological and sports performance.

## 1 Introduction

The Dictionary of Sport and Exercise Science defines anxiety as a subjective feeling of fear or threat accompanied by increased physiological activation (Anshel and Freedson, 1991). In sports psychology, competitive state anxiety (CSA) is a physical and psychological response among athletes evoked by a sports competition; CSA is accompanied by varying degrees of emotional and physical sensory experiences and is one of the most important factors affecting the outcome of a competition (Palazzolo, 2020). Martens et al. (1990a,b) categorized CSA into cognitive anxiety, somatic anxiety, and self-confidence. Cognitive anxiety involves negative thoughts and mental worries about oneself, the current situation, and potential outcomes (Morris et al., 1981). Somatic anxiety manifests as physical symptoms such as muscle tension, increased heart rate, and upset stomach. Self-confidence is similar to the concept of self-efficacy (Barnes et al., 1986) and refers to an individual’s subjective level of confidence in his/her ability, performance, and success in a competitive situation.

Goal orientation (GO) theory originated from achievement goal theory (Nicholls, 1984; Nicholls, 1989), which refers to the fact that people are oriented towards accomplishing a task (task oriented) or comparing themselves to others (ego oriented) when performing a task. People who are predominantly ego oriented can only gain a sense of accomplishment by proving themselves to be more capable than others, while those who are mostly task-directed focus on task mastery and learning for purely intrinsic reasons; they gain a sense of fulfillment from personal progress, high levels of effort, and learning new things (Ommundsen and Pedersen, 1999). The literature has documented the significance of GO in academic performance in various fields of education (Yao et al., 2021; Yao and Zhu, 2024).

Sports anxiety and GO often affect SP. The level of CSA is usually inversely related to SP, while the level of self-confidence significantly affects athletes’ SP (Kais and Raudsepp, 2004; Chamberlain and Hale, 2007; Tsopani et al., 2011). In addition, task and ego orientation in GO are correlated with SP, and a positive task orientation increases the level of SP.

Track and field (T&F) sports include walking, running, jumping, and throwing events; they constitute the foundation of human life and movement as well as the physical and technical basis of most sports. T&F sports play an important role in the training and teaching of competitive and school sports, and T&F is known as the “mother of all sports.” However, in current research, there are few studies on sports anxiety (Kirkby and Liu, 1999; Parnabas et al., 2015a,b; Gai, 2024) and GO (Tenenbaum et al., 1999; Miryousefi and Zamani, 2018) in T&F programs. Studies on both sports anxiety and GO are even scarcer regarding the correlation with SP (Hanrahan and Gross, 2005). In addition, existing studies mostly involve professional or semi-professional level athletes, and there is a lack of samples comprising college students. We conducted the present study based on the context of higher education in China and built a pathway model to explore how Chinese college athletes’ CSA and GO affect their SP. Specifically, we focused on the mediating role of GO in this process. Finally, rooted in our findings, we derived recommendations to improve teaching and training as well as to enhance SP in T&F athletes.

## 2 Literature review

### 2.1 CSA and SP

Recently, emotions have received substantial attention from scholars from various fields of education (Guan et al., 2023; Yao et al., 2023; Yao et al., 2024; Zhu et al., 2022). In the field of sports psychology, CSA has been identified as a significant psychological variable that is closely associated with SP (Cox et al., 2003). Research has consistently demonstrated that this construct can exert a profound influence on sports performance and achievement (Tsopani et al., 2011; Moreira da Silva et al., 2021). Empirical evidence suggests that athletes exhibiting lower levels of CSA tend to achieve superior outcomes compared to their counterparts with elevated anxiety levels. The latter often display diminished self-confidence, which is a critical determinant of athletic success (Chapman et al., 1997; Chamberlain and Hale, 2007; León-Prados et al., 2011). Furthermore, self-confidence has the strongest and most consistent correlation with SP (Kais and Raudsepp, 2004; Chamberlain and Hale, 2007; Tsopani et al., 2011).

However, the relationship between CSA and SP is not uniform. Some studies have reported a modest association between these variables, with cognitive and somatic anxiety demonstrating a weaker association with sports performance, while self-confidence has emerged as the most robust, consistent predictor of SP, highlighting its pivotal role in this context (Craft et al., 2003). Besides, recent studies have shown that CSA indirectly influences SP through various mediating factors. Psychological capital serves as a mediating variable, and sports anxiety impacts SP through psychological capital (Lee et al., 2022). Athletes exhibiting higher levels of mental toughness tend to display superior coping mechanisms in managing competitive anxiety, thereby leading to enhanced SP (Mahato and Thander, 2023). Individuals with elevated achievement motivation possess a greater capacity to mitigate the adverse effects of sports anxiety, which contributes to improved athletic outcomes (Németh and Balogh, 2021).

Research incorporating GO theory as a moderating variable has further elucidated the relationship between CSA and SP. GO theory can moderate the relationship between competitive cognitive anxiety and SP through the dimensions of task and ego orientation. Athletes characterized by high task orientation and low ego orientation can maintain (or even enhance) their SP under conditions of heightened anxiety (Peng and Zhang, 2021). In addition, task orientation is a significant, positive predictor of SP, whereas ego orientation reveals a weaker predictive relationship with athletic outcomes (Farkhandeh et al., 2015). While the existing studies explored the potential moderating effects of GO between CSA and SP, the mediating role of GO in this process has not been examined, particularly within the context of T&F, as illustrated in the following section.

### 2.2 GO and SP

GO theory has been extensively applied in sports psychology. Tenenbaum et al.’s (1999) investigation of middle-distance runners indicated that both ego and task orientation had moderate yet significant correlations with improvements in running performance. In a recent study, although Fernández-Río et al. (2018) did not directly examine the impact of GO on SP, they observed that as the level of athleticism increased, the achievement goals became more pronounced, which played a dominant role in settings for both training and competition.

Specifically, ego orientation can have both positive and negative effects on SP. Sari (2015) showed that ego orientation can have a detrimental impact on sports performance. However, Jung and Ahn (2017), who conducted his research among military students, indicated that ego orientation had a positive effect on SP, with its influence surpassing that of task orientation. Additionally, task orientation has consistently been positively correlated with SP in different kinds of sports, including basketball and rhythmic gymnastics (Peng and Zhang, 2021; Tsopani et al., 2011). For example, Peng and Zhang’s (2021) study of 81 college basketball players further elucidated the relationship between GO and SP. Their findings suggest that high levels of task orientation can lead to better performance of basketball players. Within the context of T&F, task orientation is also positively related to SP (Van-Yperen and Duda, 1999; Gilson et al., 1999), with high-level athletes exhibiting greater levels of task orientation and being more likely to perform at their optimal competitive level than their lower-level counterparts (Koumpoula et al., 2011).

Generally, GO and SP are significantly correlated in SP. While task orientation consistently demonstrates positive relationship with SP, inconsistent findings emerge concerning the association between ego orientation and SP. Therefore, more research is needed to further investigate the relationship between GO and SP.

### 2.3 CSA and GO

We also investigated the relationship between CSA and GO. In general, self-confidence can positively influence both ego and task orientation (Ntoumanis and Biddle, 1998; Lee et al., 2021). However, cognitive and somatic anxiety are negatively related to task orientation (Ommundsen and Pedersen, 1999; Tomczak et al., 2022). Meanwhile, the two kinds of anxiety are positively associated with ego orientation (Li and Chi, 2007).

While existing studies have explored the interconnections between CSA and GO, further research is needed to identify the differences across sports, levels of athletic proficiency, and the correlations between the three dimensions of CSA and ego and task orientation. In competitive sports, the effects of CSA on GO remain the focal points of investigation as both constructs ultimately impact athletic outcomes.

### 2.4 CSA, GO, and SP in athletics

T&F athletes are characterized by high-intensity training, advanced technology, and stringent psychological demands. Consequently, athletes’ psychological quality in this area is exceedingly high. During competitions, athletes must not only focus on their physical fitness and technical proficiency but also attend to psychological factors that significantly impact their performance. Hence, CSA and GO are critical variables that affect the performance of T&F athletes to varying degrees.

The existing literature has established the significant correlations between CSA and GO (Ntoumanis and Biddle, 1998; Lee et al., 2021), as well as between GO and SP (Peng and Zhang, 2021; Jung and Ahn, 2017). Therefore, it is reasonable to infer that GO may play a mediating role between CSA and SP. However, to the best of our knowledge, there is no empirical study that systematically examine the mediating effect of GO between the two variables. Therefore, within the context of tertiary physical education in China, our study intends to examine the interplay between three variables. The following two research questions guide the whole study: (1) using path analysis to identify the relationships among CSA, GO, and SP; and (2) exploring the mediating role of GO in this process.

## 3 Research method

### 3.1 Context and participants

We conducted this investigation during the fall semester of 2024 at a public university in Hunan Province in mainland China. The participants were 87 college students, comprising 48 males and 39 females (44.8% female), with a mean age of 19.28 years (SD = 1.12). The students were enrolled in business, computer science, and education courses. The competitive event in question was a 1,500-meter race. All athletes who completed the questionnaire were affiliated with institutions in Hunan Province and were enrolled as university students. Prior to participating, all students gave informed consent, indicating that their involvement in the study was voluntary. The study protocol was approved by the university’s institutional review board. The Institutional Review Board is responsible for ensuring that all research activities comply with relevant ethical standards and regulatory requirements to protect the rights and safety of participants.

### 3.2 Instrument

To assess the impact of motor state anxiety on SP, we adapted questions from Martens et al. (1990a,b) and Wish (1994). The scale comprises 9 items requiring students to make rapid decisions based on the options provided. For instance, “I doubt myself” represents cognitive anxiety, “I feel distracted” reflects somatic anxiety, and “I am sure of this game” signifies self-confidence. The Cronbach’s alpha coefficients indicate good internal reliability for cognitive anxiety (3 items, *α* = 0.854), somatic anxiety (3 items, α = 0.789), and self-confidence (3 items, α = 0.855). We used the 6-item Task and Ego Orientation in Sport Questionnaire, adapted from Duda and Nicholls (1992) in a study by Chinese scholars (Chen and Si, 1999). The questionnaire has good structural validity and internal reliability on scale tests. Sample items include, “I learn a movement that gives me pleasure when I learn it” (task orientation, 3 items, α = 0.882) and “I do better than my classmates when…” (ego orientation, 3 items, α = 0.824). To ensure the rationality of the questionnaire, we consulted three experts in sports psychology and physical education (including physical education teachers) regarding its overall rationality and validity. We also conducts a pilot test with expert reviewers, including experienced physical education teachers and university students, to evaluate the accuracy and clarity of the content presentation.

### 3.3 Data collection

We translated and modified the options in the original scale. Initially, the target population consisted of college students aged 18–22 years. Subsequently, we developed an unaltered version of the scale. We assembled a panel of experts with specialized knowledge in relevant fields and bilingual proficiency to determine the translation style, terminology conventions, and other key considerations. The experts then translated each item on an item-by-item basis. Next, the translated versions underwent a rigorous review process, during which we cross-examined panel members to provide suggestions for refinement. Afterward, we conducted a back-translation procedure wherein the Chinese version was retranslated into English by a separate team of bilingual experts. This ensured that the back-translated text would accurately preserve the meaning and results of the original version. We then made cultural adaptations to optimize the translation in accordance with local linguistic norms, ensuring that the final expression would align with cultural expectations and language use conventions. The questionnaire was distributed online via Questionnaire Star. Students completed it 30 min before the competition started, with classroom teachers and assistants helping with collection and completion.

Students’ scores were assessed by three certified Level 1 Athletics and Timing Statistics Judges. In cases where two timing devices displayed identical results and the third device showed a different value, we selected the time recorded by the two devices in agreement as the official outcomes. When discrepancies emerged among the three devices, the median value became the final score. We converted all recorded times into seconds to facilitate statistical analysis.

### 3.4 Data analysis

We employed SPSS 25.0 for preliminary analysis, covering descriptive statistics, Cronbach’s *α*, and bivariate correlations between key variables. Primary analysis was conducted in Mplus Version 8.3 (Muthén and Muthén, 2017) using path analysis. Maximum likelihood (ML) was employed for all parameter estimation. Using confirmatory factor analysis (CFA), we assessed the questionnaire’s structural validity by loading items related to CSA (three types) and GO (two types) into the respective factors. We examined the following fit indices for the model’s data: the chi-square statistic (*χ*^2^), the comparative fit index (CFI, good > 0.95), the Tucker-Lewis index (TLI, good > 0.95), the root mean square error of approximation (RMSEA, good < 0.06), and the standardized root mean square residual (SRMR, good < 0.05).

**Figure 1:**
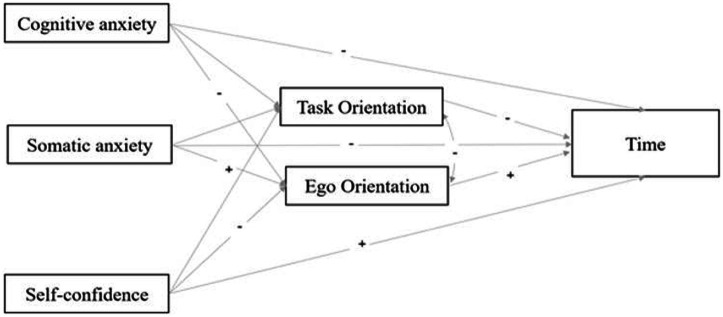
Hypothetical “competitive state anxiety – goal orientation – sports performance” model.

Based on the literature, we built a hypothetical “competitive state anxiety – goal orientation –” model (Figure 1). Cognitive and somatic anxiety were negatively correlated with ego orientation, whereas self-confidence was positively correlated with task orientation and negatively correlated with ego orientation. Thus, athletes with deeper self-confidence may have higher task orientation and lower ego orientation. Both types of GO affected SP; the task orientation scores were positively correlated with SP, whereas ego orientation negatively affected performance.

## 4 Results

### 4.1 CFA, descriptive statistics, and bivariate correlations

**Figure 2:**
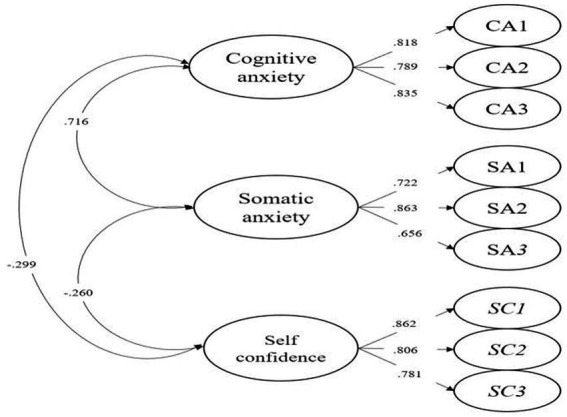
Confirmatory factor analysis model of competitive state anxiety.

To establish the structural validity of the three state anxiety constructs, we performed CFA on the selected model, which demonstrated an excellent fit to the data, as evidenced by the following indices: *χ*^2^ (24) = 23.676, *p* = 0.480, CFI = 0.999, TLI = 1.009, RMSEA [90% CI] = 0.000 [0.000, 0.086], and SRMR = 0.039. The factor loadings for the 9 items ranged from 0.656 to 0.863 (Figure 2), with the majority exceeding 0.80 and approaching 0.90. Furthermore, inter-factor correlations revealed a positive correlation between somatic and cognitive anxiety (*r* = 0.716, *p* < 0.001). In contrast, self-confidence was negatively correlated with both cognitive (*r* = −0.299, *p* < 0.001) and somatic anxiety (*r* = −0.260, *p* < 0.001).

**Figure 3:**
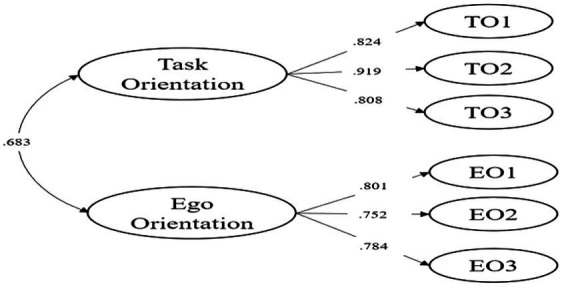
Confirmatory factor analysis model of task and ego orientation in sports.

In addition to the state anxiety constructs, we validated the CFA results for GO. The model specifies that task orientation and ego orientation items are loaded onto three distinct latent factors. The model fit indices indicated a good fit: *χ*^2^ (8) = 18.793, *p* = 0.016, CFI = 0.966, TLI = 0.986, RMSEA [90% CI] = 0.125 [0.051, 0.119], and SRMR = 0.036. The factor loadings for the 6 items ranged from 0.752 to 0.919 (e.g., Figure 3). The two motion orientation factors was positively correlated (*r* = 0.683, *p* < 0.001). Descriptive statistics and the Pearson’s correlations among the key variables are presented in Table 1.

**Table 1:** Descriptive statistics and bivariate correlations.

	Task orientation	Ego orientation	Cognitive anxiety	Somatic anxiety	Self-confidence	Time
Task orientation	–					
Ego orientation	0.761^**^	–				
Cognitive anxiety	−0.164	−0.097	–			
Somatic anxiety	−0.086	−0.042	0.837^**^	–		
Self-confidence	0.437^**^	0.351^**^	−0.248^*^	−0.209	–	
Time	−0.402^**^	−0.309^**^	0.186	0.130	−0.164	–
Mean (SD)	3.51 (0.95)	3.54 (0.91)	2.23 (0.77)	2.23 (0.92)	2.50 (0.72)	–
Skewness	−0.54	−0.55	0.10	−0.01	−0.25	–
Kurtosis	−0.066	−0.035	−0.312	−0.092	−0.419	–

^*^Correlation is significant at the 0.05 level (2-tailed).^**^Correlation is significant at the 0.01 level (2-tailed).

### 4.2 The effect of CSA on SP through task and ego orientation in sports

**Figure 4:**
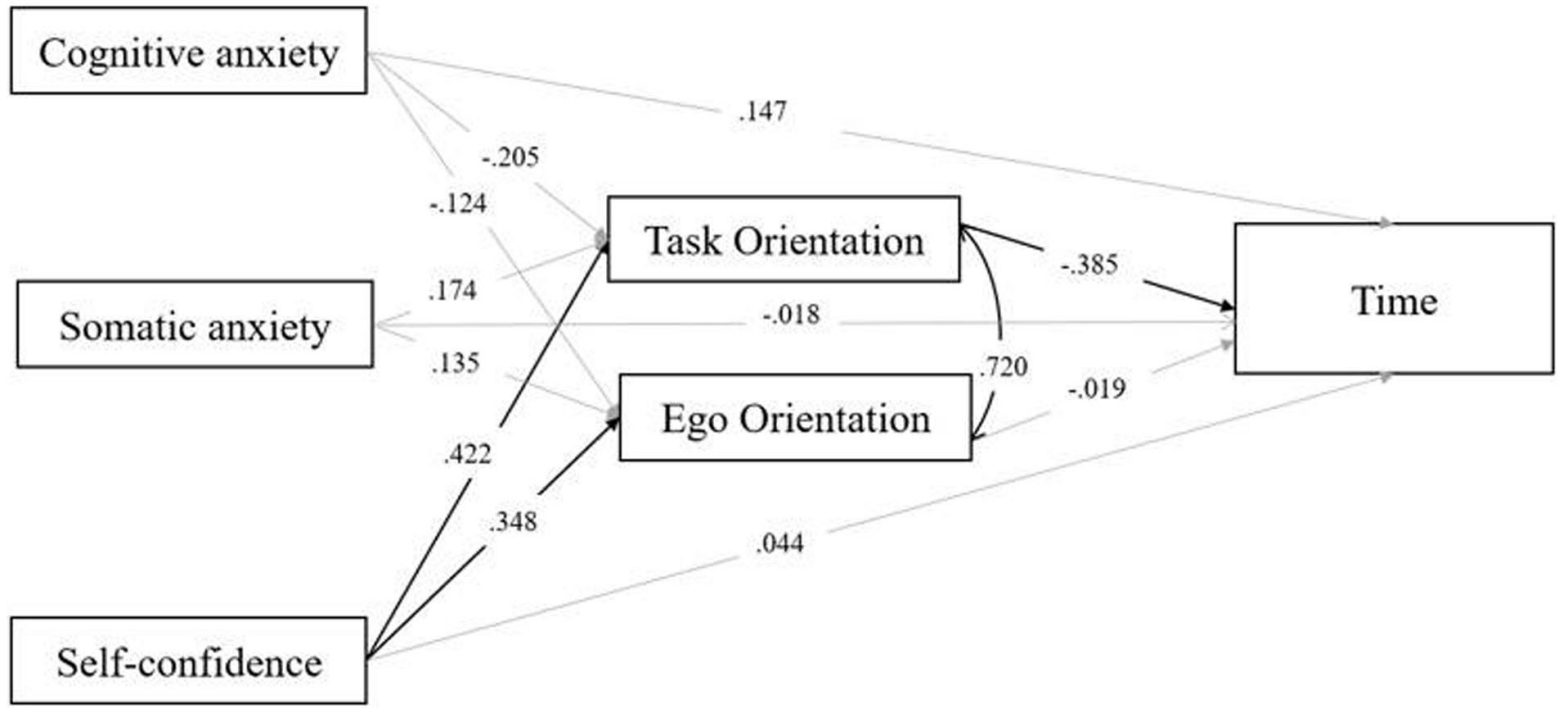
Standardized path coefficients of the model. Solid gray lines indicate non-significant paths in the model, while solid black lines denote significant paths in the model.

As shown in Table 1 and Figure 4, anxiety in all three competitive states had no significant impact on time; only task orientation significantly affected time, with a negative correlation, as evidenced by a standardized path coefficient of −0.385, *p* = 0.012. This suggests that the more students are focused on task orientation goals, the shorter their performance on the 1,500-meter run. Conversely, the level of ego orientation did not display a significant correlation with time, with a path coefficient of −0.019. Additionally, self-confidence significantly influenced the level of GO, with a path coefficient of 0.422, *p* < 0.001. Furthermore, the level of self-confidence significantly affected the level of ego orientation, with a path coefficient of 0.348, *p* < 0.001.

The R square for the dependent variable, time, was 0.178 in this model, *p* = 0.017, indicating that task orientation was the most influential variable. The R square for task orientation was 0.203, *p* = 0.011, whereas the R square for ego orientation was 0.129, *p* = 0.100. These results imply that the model explains approximately 16.2 and 20.3% of the variance in time and task orientation, respectively. Among all the mediated effects of CSA on SP, only one mediating path was significant: the path from self-confidence to task orientation time, with a standardized path coefficient of −0.162, *p* = 0.026, and 95% CI = [−0.306–0.019]. This finding denotes that task orientation significantly mediates the relationship between self-confidence and time. Specifically, students with greater levels of self-confidence exhibited higher levels of task orientation ability, which ultimately resulted in shorter durations of performance on the 1,500-meter run.

## 5 Discussion

Our primary objective was to elucidate the complex relationship between CSA and GO as well as their impact on SP among college students in mainland China. Specifically, we aimed to explore the mediating role of GO in this process. The following section provides a detailed discussion of our findings within the context of the existing literature.

### 5.1 The relationship between CSA and SP

According to the results of Pearson’s correlation and path analysis, we observed no significant correlation among the three dimensions of CSA (cognitive anxiety, somatic anxiety, and self-confidence) and SP. These results diverge from those of other studies in the literature, which have reported a significant negative relationship between CSA and SP (Kais and Raudsepp, 2004; Chamberlain and Hale, 2007; Tsopani et al., 2011). For instance, Kais and Raudsepp (2004) observed that both cognitive and somatic anxiety negatively affected the performance of beach volleyball players. Similarly, Chamberlain and Hale (2007) found that higher levels of CSA were associated with poorer performance in a golf putting task. In the present study, we did not find such a relationship among Chinese college students in T&F programs.

This discrepancy can be attributed to several factors. First, our sample consisted of college students, not professional athletes. Compared with professional athletes, ordinary college students exhibit more diverse motivations and expectations for participating in sports, with relatively lower expectations regarding competitive outcomes. Consequently, the impact of sports anxiety on performance is less pronounced (Zhang et al., 2024). In contrast, experienced professional athletes have different psychological mechanisms. Boardley et al. (2015) found that experienced athletes displayed higher levels of cognitive anxiety who may struggle to focus on the technical aspects of their performance during competitions, ultimately leading to poor SP.

Second, most prior studies have focused on the impact of anxiety levels on SP during team sports. For example, Parnabas et al. (2014) observed that, for team events professional players, cognitive anxiety tends to increase during a competitive period; further, cognitive anxiety has a more significant impact on SP (Marwat et al., 2021). In team sports, the manifestation of anxiety can significantly impair the coordination and communication among teammates, which are critical for achieving optimal performance. Anxious athletes often experience diminished interpersonal synchronization, leading to breakdowns in the fluid execution of strategic plays and collective decision-making. This disruption in team cohesion not only hinders the overall synergy required for success but also compromises the ability to adapt effectively to dynamic game situations, ultimately undermining the team’s competitive edge.

### 5.2 The effect of GO on SP

The results of Pearson’s correlation revealed that GO was significantly correlated with SP. Higher levels of task orientation are associated with shorter SP durations, a finding consistent with the current literature (Tsopani et al., 2011; Abolghasemi et al., 2013; Farkhandeh et al., 2015). Athletes with a task-oriented approach are more likely to focus on individual progress and effort, which serves to enhance SP. Additionally, the Pearson’s correlation analysis indicated that ego orientation was also significantly correlated with SP. Although this outcome is consistent with past research (Jung and Ahn, 2017), in which higher ego orientation was associated with better SP among students, this result contradicts the findings of other studies (Sari, 2015; Peng and Zhang, 2021).

Furthermore, in the path model, we observed no significant correlation between ego orientation and SP, likely due to the influence of other variables. In a regression analysis, introducing additional variables can render the relationship between two previously significantly correlated variables insignificant (Neter et al., 1996; Freedman et al., 2009). Hence, in the present study, the impact of ego orientation on SP became non-significant when we collectively considered the effects of diverse kinds of exercise anxiety and task orientation.

### 5.3 The mediating role of GO

Our findings suggest that self-confidence indirectly influences SP through task orientation. Specifically, higher levels of self-confidence lead to higher task orientation, which in turn generates better SP. This result is supported by previous studies, which found that self-confidence and GO collectively exert positive effects on SP (Ommundsen and Pedersen, 1999; Li and Chi, 2007). Self-confidence enhances task orientation by increasing athletes’ confidence in their ability to successfully complete a task. This heightened confidence motivates athletes to focus on personal progress and effort, ultimately leading to improved SP. The mediating role of task orientation implies that educators can enhance college students’ self-confidence, increase their level of task orientation, and improve their SP through specific instructional strategies, as discussed below.

### 5.4 Implications

Based on our findings, we propose the following evidence-based teaching recommendations. First, teachers and coaches should enhance student athletes’ self-confidence by implementing scientifically structured training programs (Koumpoula et al., 2011) and progressively setting challenging yet appropriate tasks according to students’ athletic abilities. This approach enables students to experience success, thereby gradually stimulating and enhancing their self-confidence (Li and Chi, 2007). Second, teachers should instruct students to establish clear and challenging goals (Locke and Latham, 2002), incorporate tools to assess self-efficacy (e.g., self-efficacy scales) into training (Biddle, 1999), and regularly monitor athletes’ confidence levels. These strategies have been shown to significantly improve athletes’ self-confidence and performance (Weinberg and Gould, 2018).

Additionally, designing diverse training content (Deci and Ryan, 1985) as well as organizing activities for students to summarize and reflect after trainings and competitions (Ericsson et al., 1993) can further enhance their self-confidence. For instance, showcasing students’ excellent performance through video replay and providing constructive feedback and encourage athletic ment through positive language strategies can help students build confidence in their abilities. Positive feedback and successful experiences are key factors for boosting self-confidence (Hanton and Connaughton, 2002). Furthermore, appropriate self-orientation positively impacts sports performance (Jung and Ahn, 2017). Hence, teachers should not overlook the role of self-orientation in their students’ sports performance and consider their development through a growth-mindset model (Diemberger, 2021). However, given that the literature has widely documented the potentially negative effects of self-orientation on sports performance (Hanrahan and Gross, 2005; Sari, 2015), teachers should also adopt a cautious, balanced approach when integrating self-orientation into regular teaching and training practices.

## 6 Conclusion

We investigated the complex relationship among CSA, GO, and SP within the context of a public university in Huna Province, China; the participants were students not majoring in physical education. This research contributes to the literature in several ways. At the theoretical level, we analyzed the effects of CSA and GO on SP, extending the study of CSA and GO to the athletic domain. By constructing a model delineating the relationships among CSA, GO, and SP, we identified task orientation as the sole mediating variable between self-confidence and SP. Overall, this study expands the existing theoretical framework of sports anxiety, GO, and SP in the field of athletics. Moreover, our findings hold significant practical value for T&F coaches and educators. The results provide specific recommendations for teaching and training practices and offer actionable insights to enhance both instructional strategies and athletic outcomes.

That said, this study has several limitations. First, the sample was small and limited to college students from one single university, which may restrict the generalizability of the findings to other populations and contexts. Future research should prioritize recruiting a more diverse sample of college students—including both professional and amateur athletes from various regions and cultural backgrounds, along with a range of athletic abilities—to determine whether the relationships among these variables vary across athletic populations. Second, due to the study’s cross-sectional design, we interpreted the observed relationships between the variables as correlations rather than causal predictions. Longitudinal studies are warranted to establish a clearer understanding of these temporal relationships. For instance, future research could measure the levels of CSA and GO at multiple time points, such as 4 weeks prior to, 1 week prior to, 1 week after, and 4 weeks following a collegiate competition. This approach would provide deeper insights into the dynamics of these constructs over time. Additionally, future research could explore other potential moderating variables (e.g., coping strategies, motivational climate, mental toughness) to gain a more comprehensive understanding of how these factors may influence the relationships among exercise anxiety, GO, and SP.

## Data Availability

The original contributions presented in the study are included in the article/supplementary material, further inquiries can be directed to the corresponding author.
